# Efficacy and Safety of Adoptive BK Polyomavirus-Reactive T-Cell Therapy for Active Infection or Disease in Transplant Recipients: A Systematic Review

**DOI:** 10.3390/v18101166

**Published:** 2026-10-20

**Authors:** Felix Bratosin, Shiva Charana Kodimala, Alexei Croitor, Vlad Dema, Ovidiu Rosca

**Affiliations:** 1 Transplant Infectious Diseases, Division of Infectious Diseases, McGovern Medical School at UTHealth Houston, Houston, TX 77030, USA; felix.bratosin@uth.tmc.edu; 2 St Mary’s Hospital, Athens, GA 30606, USA; charana.cherry8@gmail.com; 3 Discipline of Urology, Department XV, “Victor Babeș” University of Medicine and Pharmacy, 300041 Timisoara, Romania; 4 Discipline of Infectious Diseases I, Department XIII, “Victor Babeș” University of Medicine and Pharmacy, 300041 Timisoara, Romania; ovidiu.rosca@umft.ro

**Keywords:** BK virus, immunotherapy, adoptive, T-Lymphocytes, cytotoxic, hematopoietic stem cell transplantation, kidney transplantation

## Abstract

Background and Objectives: BK polyomavirus (BKPyV) causes haemorrhagic cystitis (HC) after allogeneic haematopoietic cell transplantation (HCT) and nephropathy (BKPyVAN) after kidney transplantation, with no antiviral of established efficacy. We evaluated the clinical activity and treatment-emergent safety of adoptively administered BKPyV-reactive T cells. Methods: PubMed, Europe PMC and ClinicalTrials.gov were searched to 1 September 2026 with citation checking; full-length reports of therapeutic infusion were eligible and prophylaxis-only reports excluded, following PRISMA 2020 and SWiM. Results: Twenty-two reports (about eighteen analytic datasets) were included, and overlapping publications linked and not summed. Fifteen independent cohorts contributed 241 evaluable recipients, of whom 178 (73.9%) responded by their own definitions (pooled proportion 87%, 95% CI 73 to 97; I^2^ = 70%). Response did not follow the expected phenotype gradient (69.1% HC, 70.0% DNAemia, 75.0% BKPyVAN, overlapping intervals), and organ-level outcomes diverged: haematuria or cystitis resolved in 19 of 25 (76.0%), whereas no cohort documented consistent renal recovery once nephropathy was established. In the only placebo-controlled trial, more kidney recipients achieved a week-24 viral load decline with biweekly posoleucel than placebo, but three placebo-controlled posoleucel trials after HCT, including a phase III HC trial of 97 participants, were discontinued in December 2023 for futility without safety concerns. Acute graft-versus-host disease ranged from 0% to 25% (4.0% BKPyV-restricted, 12.2% whole-cohort denominators), rejection occurred in 4 of 69 (5.8%) exposed kidney recipients versus 0 of 19 on placebo, and one fatal cytokine-release syndrome was reported. Conclusions: BKPyV-reactive T cells show reproducible virological activity and acceptable short-term tolerability, but overlapping, mostly uncontrolled evidence does not establish comparative clinical efficacy or graft benefit. Development should target persistent DNAemia after failed reduction in immunosuppression and before irreversible fibrosis.

## 1. Introduction

BK polyomavirus (BKPyV) persists in the reno-urinary epithelium of more than 80% of adults and re-emerges when T-cell function is impaired [1,2,3]. Plasma DNAemia develops in 13% and biopsy-proven nephropathy (BKPyVAN) in 8% of kidney recipients within the first year, and histological stage at diagnosis predicts irreversible graft loss [4,5]. With no antiviral of established efficacy, guidance relies on screening and staged reduction in immunosuppression [6,7,8], which controls the virus slowly, often after injury is advanced, and exposes the allograft to rejection [6,9]. No adjunctive drug has proved reliably effective [9,10].

After allogeneic HCT, the dominant manifestation is BKPyV-associated haemorrhagic cystitis (HC), reported in 13% to 17% of recipients, most often after cord-blood and HLA-mismatched grafts, and associated with prolonged hospitalisation, transfusion and, in severe grades, treatment-related mortality [11,12]. Guidance recommends supportive care, since cidofovir and other options lack convincing evidence and carry nephrotoxicity risk [13,14,15]. Both settings share insufficient BKPyV-specific cellular immunity but differ in target organ, immunosuppression and competing harms.

The e mergence of BKPyV-specific CD4+ and CD8+ T-cell responses against capsid and large T antigens accompanies the clearance of DNAemia and recovery from nephropathy [3,16,17,18], and virus-specific T cells (VSTs) have been developed as therapy for cytomegalovirus and Epstein–Barr virus after HCT [19,20]. BKPyV-reactive cells can be expanded ex vivo against defined antigens, enriched rapidly by interferon-gamma capture, or delivered within a multivirus product, and may originate from the HCT donor, a related donor or a banked, partially HLA-matched third-party source [21,22]. Because effector function is restricted through recipient-presented HLA, both antiviral efficacy and alloreactive toxicity, including graft-versus-host disease (GVHD) and allograft rejection, are relevant endpoints.

Products, doses, HLA matching and repeat-dosing rules differ, as do response definitions, assays, immunosuppression changes and follow-up, and several publications describe overlapping participants. Previous syntheses pooled BKPyV within multivirus conference data [23], predated most T-cell cohorts [15], addressed pharmacological interventions in kidney transplantation [9,10], or were narrative without PRISMA-based selection and risk-of-bias assessment [2,18,22]. No full-length systematic review restricted to therapeutic BKPyV-reactive T cells across both settings was identified.

This review evaluated efficacy-related and treatment-emergent safety outcomes of adoptively administered BKPyV-reactive T cells in adult or paediatric HCT or solid organ recipients with laboratory-confirmed BKPyV replication, HC or BKPyVAN, versus placebo or usual care where available, with attention to overlapping cohorts, the distinction between virological response and organ recovery, and uncommon immune toxicities.

## 2. Materials and Methods

### 2.1. Review Design, Protocol, and Reporting Guidelines

This review follows PRISMA 2020 [24] and, because comparative effects could not be validly combined, presents a narrative synthesis per SWiM [25]; the checklist (Table S1) and flow diagram are supplied separately. The PICO framework specified transplant recipients with active BKPyV infection or disease, adoptively administered BKPyV-reactive T cells, any or no comparator, and virological, clinical, allograft, survival and safety outcomes. The protocol was registered with the Open Science Framework (osf.io/zcwx6), with two departures: prophylaxis-only reports were excluded, and newly identified therapeutic cohorts and companion reports were added without a cap. The unit of inclusion was the full report and of synthesis the analytic cohort, so reports from one trial or programme were linked rather than assumed independent. Unavailable values are recorded as not reported (NR).

### 2.2. Eligibility Criteria

Eligible populations were adult or paediatric allogeneic HCT or solid organ recipients with active BKPyV infection or disease (BKPyV DNA in blood or urine, clinically diagnosed HC, or biopsy-supported BKPyVAN); mixed populations qualified only when such recipients could be separated. The eligible intervention was any infused T-cell product with defined BKPyV reactivity—monospecific or multivirus; donor-derived, related-donor, autologous or third-party; expanded or cytokine-captured; single or repeated—while unselected memory T-cell products were excluded. Any comparator or none and any design were eligible if at least one BKPyV-specific virological, clinical or safety outcome after infusion was reported. Prophylaxis-only administration, JC polyomavirus (JCPyV) disease without active BKPyV, in vitro or manufacturing studies, inseparable reports, reviews, editorials, conference abstracts and registry entries without a full primary report were excluded; only accessible full-length manuscripts were included.

### 2.3. Information Sources and Search Strategy

PubMed, Europe PMC and ClinicalTrials.gov were searched from inception to 1 September 2026 (full strategies in Appendix A); the MeSH terms were ‘BK Virus’, ‘Immunotherapy, Adoptive’ and ‘T-Lymphocytes, Cytotoxic’ with title-and-abstract synonyms for BK virus, BKPyV, BKV, virus-specific T cells, cytotoxic T lymphocytes, adoptive transfer, posoleucel, ALVR105 and Viralym-M, and equivalent terms in the other sources. No date, language, age or design filters were applied, and records were deduplicated by PMID then normalized DOI or title. Reference lists, cited-by links and trial registration numbers were traced for interim, subgroup or expanded-cohort reports. Embase, Scopus and Web of Science were not available.

### 2.4. Study Selection, Report Linkage, and Data Extraction

Titles and abstracts were screened and uncertain records advanced to full text, where four questions were applied in the following order: was at least one transplant recipient treated for active BKPyV infection or disease; was a product with defined BKPyV reactivity infused; was a BKPyV-specific efficacy or safety outcome separable; and was the manuscript accessible? One reviewer screened and extracted, with a second source-level audit of every included report and table entry; this was not dual-independent screening and is a limitation. Full-text exclusions were assigned one primary reason (Appendix B). Extraction captured bibliographic, design, population, phenotype, product, dose, schedule, cointervention, response definition, outcome, follow-up and adverse-event fields at the most specific reported time point. Overlapping reports were identified from trial identifiers, centres, enrolment periods, participants and authors; they remained included for their distinct information but were not added across publications (Table 1).

### 2.5. Risk-of-Bias Assessment and Evidence Synthesis

Risk of bias was assessed by design: RoB 2 for the placebo-controlled trial [48] and the relevant JBI domains for nonrandomized cohorts, series and case reports [49]; numerical scores were not used. Outcomes were grouped by setting and then phenotype. Because clinical consequences depend more on phenotype than setting, response was additionally aggregated into DNAemia without organ disease, HC and biopsy-proven BKPyVAN as crude proportions with Wilson 95% confidence intervals, restricted to single-phenotype cohorts; mixed cohorts were listed separately. Organ-level endpoints (gross haematuria resolution for HC; creatinine or estimated glomerular filtration rate for BKPyVAN) were tabulated alongside the proportions, and acute GVHD and rejection aggregated likewise for BKPyV-restricted and whole-cohort denominators. A conventional meta-analysis was not performed, because the single randomized trial used schedule-specific endpoints and the remaining evidence varied in population, product, dose, definition, follow-up and immunosuppression reduction, with several cohorts overlapping.

To display the distribution of responses rather than estimate an effect, study-level proportions with Wilson 95% confidence intervals [50] were plotted for every independent cohort with a BKPyV-specific numerator and denominator, nested reports contributing once (Appendix D). An exploratory random-effects pooled proportion was computed on the Freeman–Tukey double-arcsine scale [51] with DerSimonian–Laird variance [52] and back-transformation at the harmonic mean sample size [53]; heterogeneity used Cochran’s Q and I^2^ [54], with a sensitivity analysis restricted to cohorts of at least five recipients. Acute GVHD was displayed likewise for independent HCT reports, but differing denominators and grading precluded pooling. No outcome was imputed, no denominator reconstructed and no proportion converted across incompatible definitions.

## 3. Results

### 3.1. Study Selection

**Figure 1:**
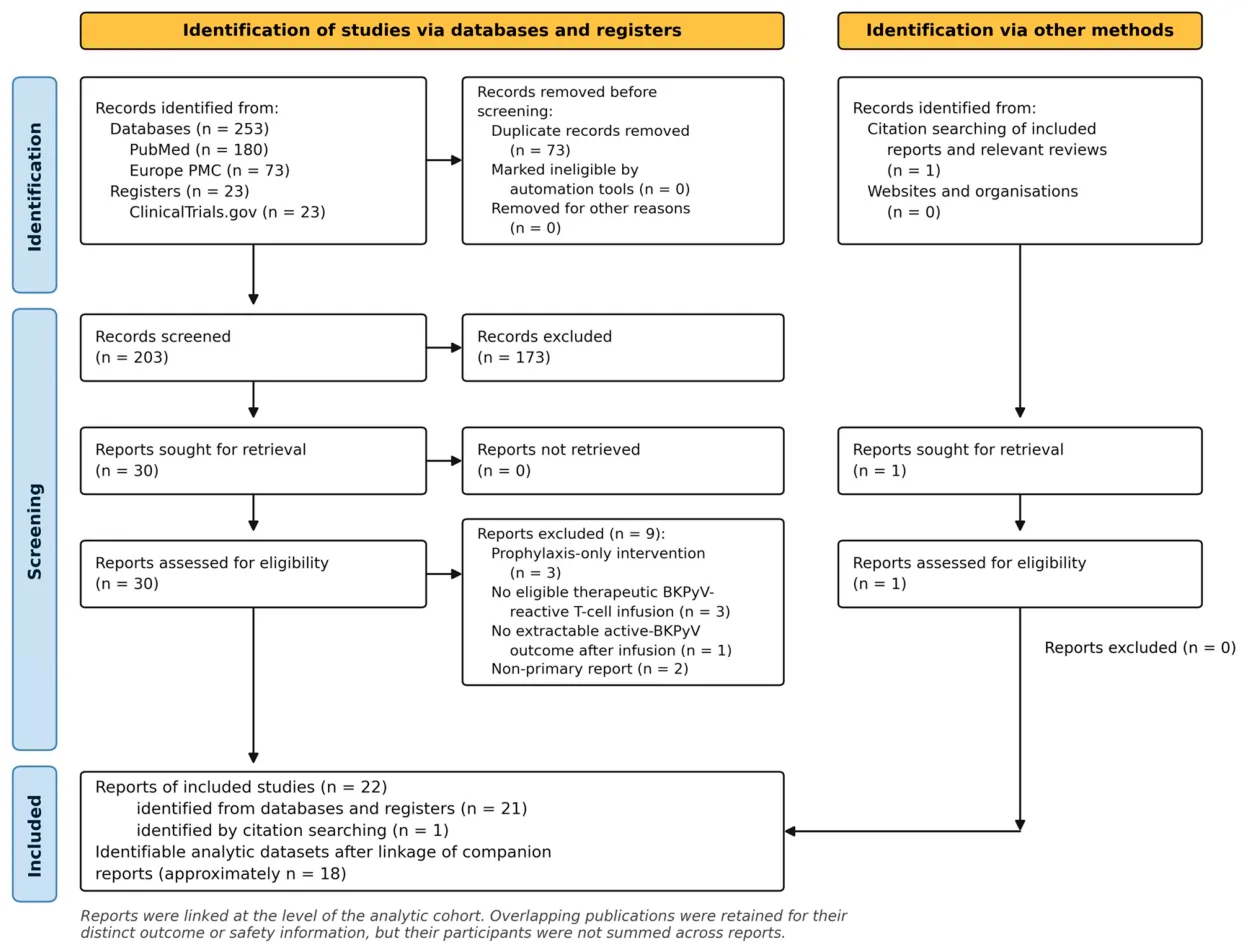
PRISMA 2020 flow diagram of the study selection.

The database searches retrieved 253 records (PubMed, *n* = 180; Europe PMC, *n* = 73) and ClinicalTrials.gov yielded 23 register records. After 73 duplicates were removed, 203 unique records were screened and 173 excluded before full text. Thirty-one reports were retrieved and ten excluded at full text: prophylaxis-only (*n* = 3), no eligible therapeutic infusion (*n* = 3), no extractable active-BKPyV outcome (*n* = 1) and non-primary report (*n* = 3). Citation checking identified one further report. Twenty-two reports describing approximately eighteen identifiable analytic datasets met the criteria, all with accessible full text (Figure 1).

### 3.2. Study Characteristics

The design, population, BKPyV phenotype, and report relationships of the 22 included reports are summarized in Table 1.

Publication years ranged from 2014 to 2026: 13 reports in allogeneic HCT only, 5 in kidney transplantation and 4 in mixed populations. The evidence comprised one randomized placebo-controlled phase II trial in kidney recipients [42], nine prospective phase I–II single-arm cohorts or their interim, expanded or subgroup analyses [26,29,30,34,35,40,43,44,45], five retrospective or observational cohorts [32,37,38,39,41] and seven case reports, small series or companion analyses [27,28,31,33,36,46,47]. BKPyV-specific denominators ranged from 1 to 59, and one report stated none [40]. Four report families were linked, Naik within Papadopoulou [26,27], Tzannou within Pfeiffer [29,30], Rubinstein 2020 within Nelson [33,34], and five reports from one Cincinnati programme with likely but unresolvable person-level overlap [34,36,39,40,41]; Jahan probably contributed to the later compassionate-access report [31,32], and the two Olson reports describe different phenotypes on the same trial [35,45]. No unique participant total was calculated.

### 3.3. Intervention Characteristics

The cellular products, sources, manufacturing methods, doses, schedules, and concomitant management are summarized in Table 2.

Multivirus products with BKPyV specificity were used in 15 reports and monospecific products in 7; sources were the HCT or organ donor in 7, third-party banks in 10, and either in 5. Manufacturing ranged from peptide-stimulated expansion over 11 to 12 days [34] to interferon–gamma cytokine capture within 48 h [46]. Doses used no common unit, spanning expanded lines at 2 × 10^7^ to 5 × 10^7^ cells/m^2^ [29,30,34,36,37,44], a fixed 4 × 10^7^ cells per posoleucel infusion [42], 1 × 10^5^ to 2 × 10^5^ CD3+ cells/kg from the BKPyV-specific bank [35,45] and about 3 × 10^2^ to 4 × 10^4^ cells/kg for cytokine-capture products [28,43,46,47]; infusions numbered 1 to 17. The reduction in immunosuppression (ROI) was reported inconsistently: an entry condition in the phase I kidney study [43], prespecified stable in a randomized-trial subgroup [42], concomitant and universal in the retrospective kidney cohort [39], permitted but not protocolized in the multicentre solid organ programme [40], and unquantified elsewhere [31]. No report stated the ROI-to-infusion interval; these and missing BKPyV-subgroup doses are recorded as NR in Table 2.

### 3.4. Efficacy-Related Outcomes

Study-specific response definitions, response proportions, virological details, clinical or graft outcomes, and follow-up are summarized in Table 3.

Among HCT reports, response proportions ranged from 3 of 4 to 27 of 27 (Table 3). The largest HC cohort reported the composite overall response of 40 of 59 (67.8%) at day 14, 43 of 57 (75.4%) at day 28 and 40 of 49 (81.6%) at day 45 [35]; the interim and expanded posoleucel cohorts reported 16 of 16 and 27 of 27 at week 6, with gross haematuria resolving in 13 of 14 and 17 of 23 evaluable HC recipients [29,30]; and the Nelson cohort reported per-infusion 4-week responses of 86% for DNAemia, 100% for HC and 87% for both [34].

In kidney transplantation, the randomized trial reported a week-24 decline of at least 1 log in 10 of 20 on biweekly posoleucel, 6 of 20 on monthly and 5 of 18 on placebo, and of at least 50% in 17 of 20, 11 of 20 and 10 of 18; clinical and graft endpoints were exploratory [42]. Uncontrolled kidney cohorts reported decline in 17 of 17 with concomitant immunosuppression reduction [39], 3 of 3 in phase I [43] and 5 of 6 with biopsy-proven BKPyVAN [45], in each case without consistent renal function improvement, while the single advanced case cleared tissue viral antigen but lost the graft to fibrosis [31]. Mixed or expanded programmes reported 45% with an unstated denominator [40] and 7 of 11 evaluable cases [32].

### 3.5. Treatment-Emergent Safety Outcomes

Treatment-emergent safety observations are summarized in Table 4.

Across the 22 reports, an acute infusion reaction or cytokine release syndrome (CRS) was described in 1, absent in 17 and not assessable in 4; the single event was severe CRS after a third infusion in a paediatric recipient with catastrophic HC, sinusoidal obstruction syndrome and critical illness, followed by multiorgan failure and death on day +63 [36]. Post-infusion acute GVHD was described in 7 reports, absent in 10 and not assessable in 5, with per-report numerators from 1 of 11 to 13 of 58 and most events grade I–II skin disease (Table 4). Allograft rejection was described in 2 reports, absent in 4 and not addressed in 16 (13 HCT-only): 3 centrally read episodes in the posoleucel groups and none on placebo, none judged treatment related [42], and one grade >1B T-cell-mediated rejection with one membranoproliferative glomerulonephritis of uncertain causality [39]. Deaths occurred in 3 of 7 recipients in one observational HCT cohort [38] and in the fatal CRS case [36]; other reports did not attribute deaths to therapy or report survival.

### 3.6. Quantitative Synthesis

**Figure 2:**
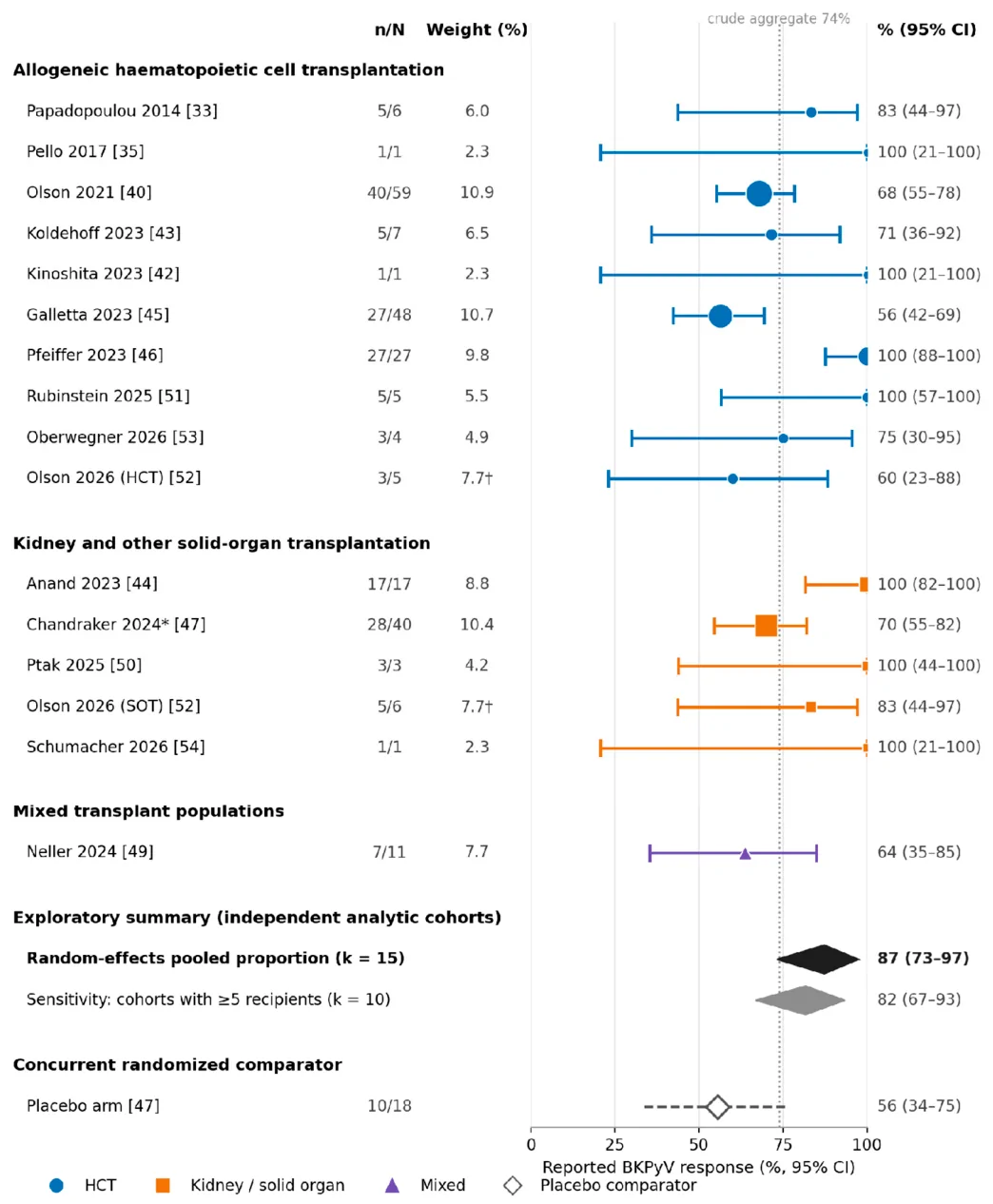
Study-level BKPyV response proportions with Wilson 95% confidence intervals, by transplant setting. Circles denote allogeneic HCT cohorts, squares kidney or other solid-organ cohorts, the triangle a mixed transplant population, filled diamonds the exploratory random-effects pooled estimates and the open diamond the concurrent placebo comparator; marker area is proportional to the number of evaluable recipients. * Posoleucel arms combined. † Olson 2026 [45] entered the pooled estimate once as 8/11 and is displayed split by transplant type. Cohorts shown: Papadopoulou 2014 [26], Pello 2017 [28], Olson 2021 [35], Koldehoff 2023 [38], Kinoshita 2023 [37], Galletta 2023 [41], Pfeiffer 2023 [30], Rubinstein 2025 [44], Oberwegner 2026 [46], Olson 2026 [45], Anand 2023 [39], Chandraker 2024 [42], Ptak 2025 [43], Schumacher 2026 [47] and Neller 2024 [32]. BKPyV, BK polyomavirus; CI, confidence interval; HCT, haematopoietic cell transplantation; SOT, solid-organ transplantation.

Of the independent cohorts, 15 contributed 241 evaluable recipients, of whom 178 (73.9%) were responders by their own definitions, with proportions from 56% [41] to 100% in seven cohorts, five of fewer than six recipients (Figure 2). The exploratory random-effects pooled proportion was 87% (95% CI 73 to 97; Q = 46.2, df = 14, *p* < 0.001; I^2^ = 70%), falling to 82% (95% CI 67 to 93) among the ten cohorts with at least five recipients, exceeding the crude aggregate by 13 points because weighting compresses cohort size. The placebo arm reached 56% (95% CI 34 to 75) on its own week-24 endpoint [42]. In Figure 2, marker area is proportional to evaluable recipients, response definitions differ (Appendix D), and the pooled diamond is descriptive rather than a treatment-effect estimate.

**Figure 3:**
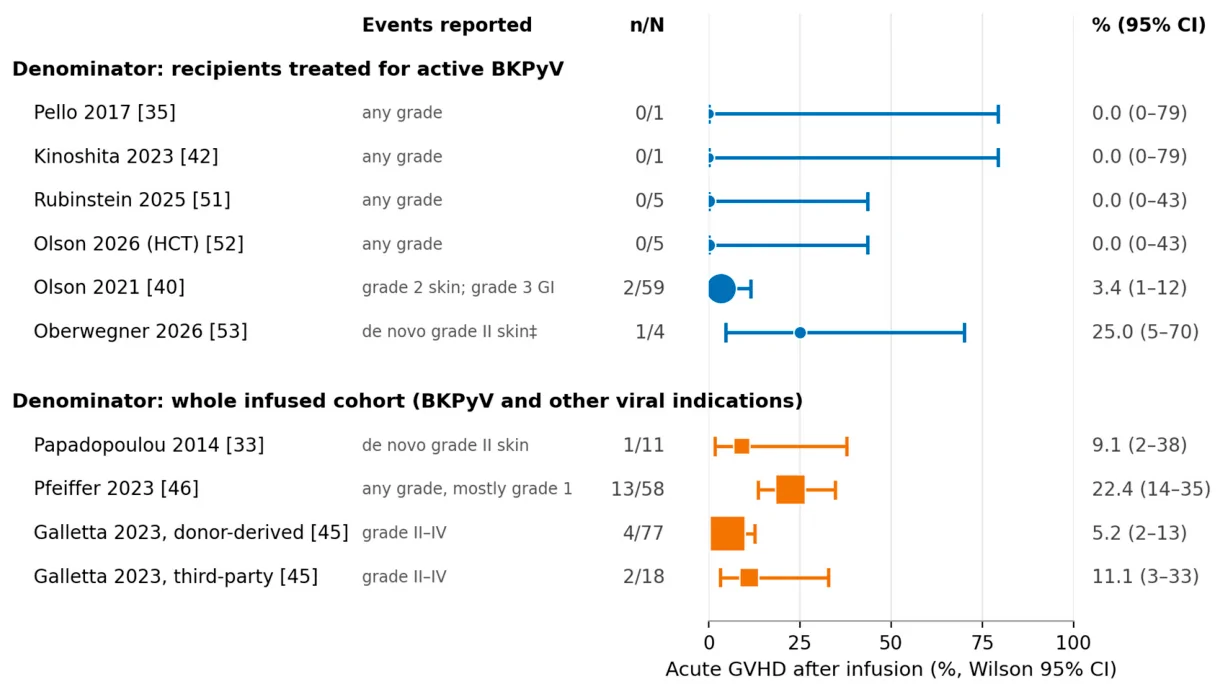
Study-level acute GVHD proportions after BKPyV-reactive T-cell infusion, with Wilson 95% confidence intervals, by denominator type. Circles denote reports with BKPyV-treated denominators and squares reports using the whole infused cohort; marker area is proportional to the denominator. ‡ One further recipient had worsening of pre-existing gastrointestinal GVHD (2/4 new or worsening). Reports shown: Pello 2017 [28], Kinoshita 2023 [37], Rubinstein 2025 [44], Olson 2026 [45], Olson 2021 [35], Oberwegner 2026 [46], Papadopoulou 2014 [26], Pfeiffer 2023 [30] and Galletta 2023 [41]; nested or overlapping reports (Naik 2016 [27], Tzannou 2017 [29], Rubinstein 2020 [33], Nelson 2020 [34]) and reports without a stated numerator or denominator (Holland 2021 [36], Koldehoff 2023 [38]) were excluded. BKPyV, BK polyomavirus; CI, confidence interval; GI, gastrointestinal; GVHD, graft-versus-host disease; HCT, haematopoietic cell transplantation.

Ten independent allogeneic HCT reports stated both an acute GVHD numerator and denominator (Figure 3): 0 of 1, 0 of 1, 0 of 5, 0 of 5, 2 of 59 and 1 of 4 in the six with BKPyV-specific denominators [28,35,37,44,45,46], and 1 of 11, 13 of 58, 4 of 77 and 2 of 18 in the four using the whole infused cohort [26,30,41]. Confidence intervals were wide below 20 recipients and overlapped across groups, so no pooled estimate was calculated. Nested or overlapping reports (Naik 2016 [27], Tzannou 2017 [29], Rubinstein 2020 [33], Nelson 2020 [34]) and reports without a stated GVHD numerator or denominator (Holland 2021 [36], Koldehoff 2023 [38]) are excluded from Figure 3, and grading thresholds differ between reports.

### 3.7. Outcomes by BKPyV Phenotype

Response across cohorts confined to a single phenotype (Table 5) was 47 of 68 (69.1%; 95% CI 57 to 79) for HC [28,35,37,38], 28 of 40 (70.0%; 95% CI 55 to 82) for DNAemia without organ disease on the week-24 at-least-50-decline threshold in the combined posoleucel arms [42], and 9 of 12 (75.0%; 95% CI 47 to 91) for biopsy-proven BKPyVAN [45,47]. The intervals overlapped substantially and did not follow the DNAemia-to-nephropathy gradient the biology would predict; the placebo arm reached 10 of 18 (55.6%; 95% CI 34 to 75) on the same endpoint [42].

The phenotypes separated only at organ level. Gross haematuria or cystitis resolved in 19 of 25 (76.0%; 95% CI 57 to 89) evaluable HC recipients across independent reports [28,30,37] and 13 of 14 in the nested interim cohort [29]. By contrast, no report documented consistent renal recovery after established BKPyVAN: creatinine did not improve despite a 72.7% virological response [45], the mean estimated glomerular filtration rate fell from 37.3 to 33.5 mL/min/1.73 m^2^ [43], the single advanced case progressed to dialysis and nephrectomy with 80% fibrosis despite near-complete antigen clearance [31], graft function stayed reduced though stable in the paediatric coinfected case [47], and recovery was explicitly not demonstrated in the multicentre solid organ program [40].

Eight further cohorts reported only a combined numerator across phenotypes and were not apportioned (Appendix D). Because these hold most of the recipients in whom DNAemia and HC coexisted, the phenotype-stratified aggregation rests on a minority of the evidence and describes what the literature permits rather than comparing phenotypes.

### 3.8. Aggregate Treatment-Emergent Toxicity

Aggregating Table 4, new-onset acute GVHD after infusion occurred in 3 of 75 recipients (4.0%; 95% CI 1 to 11) across the six independent HCT reports with BKPyV-restricted denominators [28,35,37,44,45,46] and in 20 of 164 (12.2%; 95% CI 8 to 18) across the four using the whole infused cohort [26,30,41]. The two aggregates are not interchangeable: the higher value reflects denominator composition, not greater toxicity. No grade-resolved aggregate is possible, because the 13 events among 58 recipients of the expanded posoleucel cohort were described only as mostly grade 1; where grade was stated, 10 events were grade II or higher [26,35,41,46].

Allograft rejection after infusion occurred in 4 of 69 kidney or solid organ recipients with explicit denominators (5.8%; 95% CI 2 to 14): three centrally read episodes among 42 posoleucel recipients and one grade >1B T-cell-mediated rejection among 17 in the retrospective kidney cohort, against 0 of 19 placebo recipients; none was judged treatment related [39,42]. An infusion reaction or CRS was explicitly absent in 17 reports and in all 61 randomized trial participants. The discontinued phase III HC trial prespecified acute GVHD and CRS as secondary safety outcomes, its registry record shows no excess of acute GVHD with posoleucel, and the sponsor reported no data safety monitoring board safety concern [55,56].

### 3.9. Risk of Bias and Certainty of Evidence

The randomized posoleucel trial had blinding, placebo control, prespecified schedules and objective viral load measurement, with RoB 2 concerns for late attrition and multiple exploratory endpoints [42]. The nonrandomized cohorts carried serious confounding, as immunosuppression reduction, supportive care, immune recovery and repeat dosing were uncontrolled, and the matched comparison in the largest HC cohort reduced but did not remove this [35]. Selection risk was substantial in compassionate-access cohorts and the phase I kidney study [32,43]; composite definitions created measurement concerns [29,30,34]; case reports carried very high selection and publication bias [28,31,36,46,47]; and the donor-derived versus third-party comparison was limited by nonrandom allocation, mixed indications and overlap [41]. Judgments are in Appendix C. Certainty was low for the randomized virological comparison and very low for symptom, graft, survival and rare safety outcomes.

## 4. Discussion

This review found a reproducible signal of BKPyV virological activity after adoptive transfer, generally acceptable short-term tolerability, and one placebo-controlled trial in which the more frequent posoleucel schedule produced a larger week-24 viral load decline than placebo. Three findings qualify it: heterogeneity was substantial and the placebo arm itself reached 56%; HC resolution after HCT was the only consistent clinical benefit, whereas renal recovery after established BKPyVAN was not; and the randomized phase III programme was discontinued for futility.

The contrast between settings has a biological basis. HC is a mucosal disease in which replication and symptoms are coupled, and haematuria resolved within weeks in every large HC cohort [29,30,35]. In BKPyVAN, tubular injury, inflammation and fibrosis are usually established at diagnosis and Banff stage predicts graft outcome [5], so clearing circulating virus cannot reverse structural damage, consistent with endogenous immunity predicting recovery mainly before advanced injury [16]. The graft lost despite near-complete antigen clearance [31] and the subgroup whose response was not matched by creatinine improvement [45] show this ceiling, whereas the series reporting preserved function lacks a counterfactual [43,47]. Benefit is most plausible for persistent DNAemia or early nephropathy, which the randomized trial is consistent with but did not test [42].

The randomized trial needs careful interpretation: safety was its primary objective, the virological comparison secondary with wide intervals, the placebo group also declined substantially, the difference was clearest in the prespecified stable immunosuppression subgroup, and the investigators and an editorial noted short follow-up and insufficient power for clinical outcomes [42,57]. The placebo response is unsurprising, since immunosuppression reduction alone clears DNAemia in most kidney recipients over months [6,8], which is why uncontrolled proportions cannot be read as treatment effects. The same caution applies after HCT, where immune reconstitution, tapering and supportive care all improve HC over time [13].

The most consequential development since these cohorts were published is the failure of the randomized phase III programme for posoleucel, the product that contributed the largest share of prospective evidence in this review. Three double-blind, placebo-controlled trials after allogeneic HCT were discontinued in December 2023 after pre-planned futility analyses: a phase III trial for the treatment of virus-associated HC, which enrolled 97 participants, of whom 86 (48 assigned to posoleucel and 38 to placebo) had BKPyV viruria at baseline and constituted the BK intent-to-treat population, and whose primary endpoint was time to resolution of macroscopic haematuria (NCT04390113) [55]; a phase II/III multivirus prevention trial of 451 participants (NCT05305040) [58]; and a phase III adenovirus-treatment trial of 57 participants (NCT05179057) [59]. In each, the data safety monitoring board judged the primary endpoint unlikely to be met, with no safety concern raised [56]. None had a published full-length report, so all were recorded among register records rather than included studies, yet bear on everything included. The HC trial tested under randomization the endpoint the uncontrolled HC cohorts reported most consistently, and showed no benefit. The reproducible improvement in single-arm cohorts must therefore be read as compatible with the natural history of HC rather than a treatment effect, and the phase II kidney signal [42] regarded as unconfirmed rather than established therapy.

Attributing viral clearance to adoptive therapy rather than ROI is the central inferential problem in the solid organ literature, and the included reports do not resolve it. ROI alone clears DNAemia in most kidney recipients, though over months not weeks [6,8], so for any uncontrolled cohort the question is whether the virus cleared faster than ROI alone would have. Permitting that judgment would require ROI timing relative to infusion, the direction of immunosuppressive exposure, the pre-infusion viral load trajectory, and a comparator given ROI without infusion; no report supplied all four or stated the ROI-to-infusion interval. Three partial safeguards exist: the phase I kidney study enrolled only recipients of which the DNAemia had persisted despite prior ROI [43]; the randomized trial’s prespecified stable-immunosuppression subgroup is the only design isolating the cells from concurrent ROI [42]; and ROI was universal and concomitant in the retrospective cohort where all 17 responded by week 12, so its 100% response is not a treatment effect [39]. The placebo arm quantifies the background directly—10 of 18 with at least a 50% decline and 5 of 18 with at least a 1-log decline by week 24 [42]—and because that matches every phenotype-specific proportion in Section 3.7, none of the uncontrolled proportions here can be attributed to the infused cells.

Heterogeneity across cohorts is largely a matter of design, not true differences in product activity: response definitions ranged from any viral load decrease to symptom-containing composites, time points from day 14 to 12 months and doses across five orders of magnitude, while HLA restriction, infusion number and immunosuppression changes were rarely standardized, and several publications arose from the same programmes, a recognized source of inflated precision [60]. Random-effects weighting compresses cohort size, so each single-patient report carried about a fifth of the largest cohort’s weight; with the known problems of double-arcsine back-transformation [53], the pooled proportion describes the literature rather than a response rate, and the fall from 87% to 82% suggests a modest small study effect.

The safety findings are broadly consistent with the wider VST literature, in which banked third-party products caused no immediate infusion-related events, with de novo GVHD in 2 of 50 [21] and acute GVHD in 2 of 30 despite a median HLA match of two of six [61]; reviews identify GVHD and CRS as the toxicities needing surveillance [20,62]. Here the acute GVHD proportions overlapped background rates after allogeneic HCT and could not be attributed to the product, as no cohort had an untreated comparator and denominators and grading differed. The fatal CRS case remains a signal despite confounding by catastrophic HC, sinusoidal obstruction syndrome and critical illness [36], and consensus grading would standardize reporting [63]. Rejection is a concern because cells are infused during immunosuppression reduction, so the numerical excess of centrally read episodes in the posoleucel arms, though consistent with chance, argues for protocolized biopsy surveillance [42,57].

Previous syntheses do not resolve these questions: the conference abstract meta-analysis of multivirus VSTs reported high aggregate responses without separating BKPyV-specific outcomes or addressing overlap [23], and narrative reviews lacked formal selection [18,22]; systematic reviews stressed the absence of a reliable pharmacological solution [9,10,15], and guidance centres on screening, immunosuppression reduction and supportive care [6,13]. Overlap is concentrated in a few linked families—Papadopoulou–Naik, Tzannou–Pfeiffer, Nelson–Rubinstein and the shared Cincinnati programme—which inflate precision if treated as independent. The same platforms have nonetheless reached late-phase development, with tabelecleucel in post-transplant lymphoproliferative disease [64], posoleucel in a phase 2 prophylaxis trial [65] and BKPyV-reactive products infused for JCPyV-associated progressive multifocal leukoencephalopathy [66]; comparative evidence of clinical benefit is what remains lacking.

### Proposed Patient Selection Framework and Implications for Trial Design

That virological response is broadly similar across phenotypes, whereas organ recovery is not, implies that the determinant of benefit is whether the organ can still recover, not whether the virus can be cleared. Because established nephropathy with advanced fibrosis limits recovery even after viral eradication [5,31], the therapeutic window lies after ROI has proved insufficient or unsafe and before fibrosis is irreversible. Table 6 sets out a staged framework consistent with this evidence, indicating where therapy should be considered within trials and where avoided. It is a hypothesis for trial design, not a treatment recommendation, because no stage is supported by comparative evidence of benefit, and the one stage tested under randomization, refractory HC, yielded a futile trial.

The trial design follows the same logic. The population with the greatest prospect of benefit is stage 2—persistent DNAemia after documented ROI failure and before irreversible fibrosis—enrolled with a mandatory baseline biopsy to exclude advanced fibrosis and stratify by fibrosis stage. Immunosuppression should be protocolized and held stable from randomization [42]; where ROI is unavoidable it should be prespecified, identical between arms and reported as a time-varying covariate. The comparator should be placebo on guideline-based care, given the placebo response here. The primary endpoint should be a composite that virological change alone cannot satisfy—for example, sustained DNAemia clearance at 24 weeks with freedom from a 30% eGFR decline at 12 months—with surveillance biopsy, at least 12 months of follow-up, prespecified attribution windows for CRS, GVHD and rejection [63], and power for the graft endpoint. For HC, where a phase III trial already proved futile, a further trial would need a different population, schedule or endpoint less prone to spontaneous resolution.

Clinically, these data support trial enrolment or carefully governed compassionate use for refractory BKPyV disease, with explicit monitoring for CRS, GVHD, rejection, renal function and virological response, but not replacement of guideline-based screening and immunosuppression management, nor routine treatment. For research, kidney trials should distinguish persistent DNAemia from established nephropathy, stratify by baseline fibrosis and function, and report standardized viral load and patient-important graft outcomes [67], while HCT trials should harmonize definitions of HC grade, symptom resolution, transfusion, obstruction and recurrence. Protocols should prespecify immunosuppression changes, cointerventions, release criteria, HLA restriction, dose, repeat infusion rules and attribution windows [63]. Where randomization is not feasible, prospective registries with contemporaneous controls and complete denominators would be more informative than isolated positive cases.

## 5. Limitations

The main limitations arise from the evidence base. Most reports were uncontrolled early-phase cohorts, small series or case reports, favourable cases are preferentially published, and the single placebo-controlled trial had follow-up too short for graft benefit. Population, product, dose, HLA matching, repeat dosing, response definition, cointerventions and follow-up varied substantially; several publications arose from the same programmes without resolvable overlap; and virological decline was reported far more consistently than renal function, graft survival or late toxicity. The pooled proportion combines non-equivalent definitions and the safety aggregates combine denominators of different composition, so neither estimates efficacy or toxicity. Review-level limitations include omission of Embase, Scopus, Web of Science and CENTRAL, the accessible-full-text requirement, single-reviewer screening with a source-level audit, and no author contact to resolve NR data. Because no report stated the ROI-to-infusion interval, delayed ROI-driven clearance cannot be separated from treatment effect. Finally, the three discontinued posoleucel trials exist only as registry records and a sponsor announcement, so the most relevant controlled evidence could not be appraised at full-text level.

## 6. Conclusions

Adoptive BKPyV-reactive T-cell therapy shows virological activity and generally acceptable short-term tolerability, with the clearest clinical signal in BKPyV-associated HC after HCT. Evidence for preservation or recovery of kidney allograft function remains uncertain, particularly after established nephropathy; rare immune toxicities cannot be quantified; and routine use cannot be recommended. The discontinuation of three placebo-controlled posoleucel trials for futility, including a phase III HC trial, indicates that the consistency seen in uncontrolled cohorts has not translated into demonstrable benefit under randomization. Development should concentrate on persistent DNAemia after failed immunosuppression reduction and before irreversible fibrosis, using endpoints that capture organ function rather than viral load alone.

## Figures and Tables

**Table 1:** Characteristics of the included reports and analytic cohorts.

Report	Country; Design	Period	Setting, *n*	Phenotype, *n* ^c^	BKPyV-Treated, *n*/*N* Infused ^a^	Relation to Other Reports
Papadopoulou 2014 [26]	USA; prospective ph I/II, single-arm	NR	Allo-HCT	Mixed: HC + DNAemia	6/11	Index cohort; 1 later episode excluded
Naik 2016 [27]	USA; companion case series	NR	Allo-HCT (PID)	Mixed, NS	2/NR	Within Papadopoulou 2014 [26]
Pello 2017 [28]	UK; case report	NR	Allo-HCT (haplo)	HC (severe, refractory)	1/1	Independent
Tzannou 2017 [29]	USA; prospective ph II, interim	NR	Allo-HCT	Mixed: HC 14, nephritis 2	16/38	Within Pfeiffer 2023 [30]
Jahan 2020 [31]	Australia; case report	NR	Kidney	BKPyVAN (advanced fibrosis)	1/1	Possible overlap: Neller 2024 [32]
Rubinstein 2020 [33]	USA; companion cohort	NR	Allo-HCT	DNAemia	6/NR (14 infusions)	Within Nelson 2020 [34]
Nelson 2020 [34]	USA; prospective ph I	2017–2019	HCT 38; SOT 3	Mixed: DNAemia and/or HC	41/41	Cincinnati; overlap likely, unresolved
Olson 2021 [35]	USA; ph II single-arm + matched comparison	2015–2019	Allo-HCT	HC	59/59	NCT02479698, HC cohort
Holland 2021 [36]	USA; case report	NR	Allo-HCT (paed., DOCK8)	HC (severe, catastrophic)	1/1	Within Nelson protocol; unresolved
Kinoshita 2023 [37]	USA; retrospective cohort	NR	HCT (paed., SCD)	HC (+ CMV)	1/13	Independent; 2 prophylaxis-only excluded
Koldehoff 2023 [38]	Germany; observational cohort	NR	Allo-HCT	HC	7/17 ^b^	Independent
Anand 2023 [39]	USA; retrospective, single centre	2021–2022	Kidney	Mixed: DNAemia 17, BKPyVAN 7	17/23	Within Khoury 2024 (likely) [40]
Galletta 2023 [41]	USA; retrospective comparative cohort	2017–2021	HCT (paed./young adult)	Mixed, NS	48/145	Cincinnati; partial overlap likely
Pfeiffer 2023 [30]	USA; prospective ph II, expanded	NR	Allo-HCT	Mixed: HC 25, nephritis 2	27/58	Contains Tzannou 2017 [29]
Chandraker 2024 [42]	USA; multicentre RCT, double-blind, placebo-controlled ph II	NR	Kidney	DNAemia	61/61	Independent (NCT04605484)
Khoury 2024 [40]	USA; multicentre open-label ph II	NR	SOT	Mixed, NS (incl. BKPyVAN)	NR/98	Cincinnati; overlap likely
Neller 2024 [32]	Australia/NZ; retrospective, compassionate access	NR (15 y)	HCT 12; kidney 4	Mixed, NS	16/78	Possible overlap: Jahan 2020 [31]
Ptak 2025 [43]	USA; prospective ph I	NR	Kidney	Mixed: DNAemia + BKPyVAN	3/3 (27 screened)	Independent
Rubinstein 2025 [44]	USA; prospective cohort	2022–2025	HCT	Mixed: DNAemia and/or HC	5/25	Nelson protocol; later enrolment
Olson 2026 [45]	USA; ph II subgroup	NR	Kidney 6; HCT 5	BKPyVAN	11/11	NCT02479698, BKPyVAN subgroup
Oberwegner 2026 [46]	Germany; case series	NR	Allo-HCT (paed.)	Mixed, NS (HC in 1)	4/4	Independent
Schumacher 2026 [47]	Germany; case report	NR	Kidney (paed., living donor)	BKPyVAN (+ JCPyV)	1/1	Independent

^a^*n*, recipients treated for active BKPyV infection or disease; *N*, all recipients infused for any indication. ^b^ Seven VST-treated recipients among 17 with BKPyV-HC. ^c^ Phenotype categories: DNAemia (replication without organ disease), HC (clinically diagnosed haemorrhagic cystitis) and BKPyVAN (biopsy-proven nephropathy, or nephritis where the source used that term). Reports spanning more than one category are labelled Mixed, with component numbers where stated; Mixed, NS denotes no separable phenotype. Abbreviations: allo-HCT, allogeneic haematopoietic cell transplantation; BKPyV, BK polyomavirus; BKPyVAN, BKPyV-associated nephropathy; CMV, cytomegalovirus; DOCK8, dedicator of cytokinesis 8; haplo, haploidentical; HC, haemorrhagic cystitis; HCT, haematopoietic cell transplantation; JCPyV, JC polyomavirus; NCT, ClinicalTrials.gov identifier; NR, not reported; NS, not separately stated; NZ, New Zealand; paed., paediatric; ph, phase; PID, primary immunodeficiency; RCT, randomized controlled trial; SCD, sickle-cell disease; SOT, solid organ transplantation; VST, virus-specific T cell.

**Table 2:** BKPyV-reactive T-cell product and administration characteristics.

Report	Specificity (Viruses, *n*)	Source; HLA Match	Manufacture	Dose per Infusion	Infusions, *n*	Concomitant Management
Papadopoulou 2014 [26]	Multivirus (5)	Donor; matched	Peptide expansion	0.5–2 × 10^7^/m^2^	1	Refractory infection; supportive care
Naik 2016 [27]	Multivirus (5)	Donor; matched	Expansion	1–2 × 10^7^/m^2^	1	NR
Pello 2017 [28]	BKPyV-mono (1)	Donor (haplo); matched	IFN-γ capture	0.34 × 10^4^/kg	1	After failed conventional care
Tzannou 2017 [29]	Multivirus (5)	Third-party; partial	Expansion	2 × 10^7^/m^2^	1 (repeat allowed)	Refractory post-HCT infection
Jahan 2020 [31]	BKPyV-reactive (1)	Donor (haplo); NR	Expansion	3.6 × 10^7^ total	10 (weekly)	IS modification; prior therapies
Rubinstein 2020 [33]	Multivirus (NR)	Third-party; partial	Expansion	≤5 × 10^7^/m^2^	Repeated	Eculizumab near VST dosing
Nelson 2020 [34]	Multivirus (NR)	Donor or third-party; partial	Peptide expansion, 11–12 d	≤5 × 10^7^/m^2^	1–10 (median 1)	Persistent BKPyV after HCT or SOT
Olson 2021 [35]	BKPyV-mono (1; 5 antigens)	Third-party (26 donors); partial	Expansion	1–2 × 10^5^ CD3+/kg	1–5 (≥28 d apart)	Supportive care for HC
Holland 2021 [36]	Multivirus (4)	Third-party; partial	Expansion	5 × 10^7^/m^2^	3 (2 pre-, 1 post-HCT)	Multimodal supportive care
Kinoshita 2023 [37]	Multivirus (6)	Donor; matched	Expansion	2 × 10^7^/m^2^	1 (day +105)	Concurrent CMV treatment
Koldehoff 2023 [38]	Mono- or multivirus (NR)	Donor or third-party; NR	Peptide expansion	NR	1–3	Persistent HC after HCT
Anand 2023 [39]	Multivirus (NR)	Third-party; partial	Expansion	NR	2 (median)	IS reduction 17/17
Galletta 2023 [41]	Multivirus (NR)	Donor or third-party; partial	Expansion	≤5 × 10^7^/m^2^	1–8	NR
Pfeiffer 2023 [30]	Multivirus (6; posoleucel)	Third-party; partial	Expansion	2 × 10^7^/m^2^	1 (repeat allowed)	Refractory post-HCT infection
Chandraker 2024 [42]	Multivirus (6; posoleucel)	Third-party; partial	Expansion	4 × 10^7^ fixed	3 weekly, then q14d or q28d to wk 12	Stable IS subgroup prespecified; placebo-controlled
Khoury 2024 [40]	Multivirus (NR; BK-targeted)	Third-party; partial	Expansion	NR	2 (median; 181 in 98)	IS changes allowed
Neller 2024 [32]	Multivirus (NR)	Autologous or allogeneic; varied	Peptide expansion	4 × 10^7^ (adults); 2 × 10^7^/m^2^ (children)	2–10	Compassionate access, 19 centres
Ptak 2025 [43]	BKPyV-mono (1)	Compatible donor; NR	IFN-γ capture	0.03–0.19 × 10^4^ CD3+/kg	1	Persistent DNAemia despite IS reduction
Rubinstein 2025 [44]	Multivirus (4)	Third-party; predicted restriction	Expansion	5 × 10^7^/m^2^	1–2 (≥21 d apart in 2/5)	Therapeutic use after HCT
Olson 2026 [45]	BKPyV-mono (1)	Third-party; partial	Expansion	1–2 × 10^5^ CD3+/kg (retreatment 2 × 10^6^)	2–17 (kidney); 2–6 (HCT)	Biopsy-proven nephropathy
Oberwegner 2026 [46]	BKPyV-mono (1)	Donor or third-party parent; NR	IFN-γ capture, ≤48 h	0.5–3.2 × 10^4^ CD3+/kg	1–3	Refractory paediatric infection
Schumacher 2026 [47]	BKPyV-mono (1; VP1/LT)	Living kidney donor; partial	IFN-γ capture	1.0 then 3.9 × 10^4^/kg	9 (q4w)	JCPyV coinfection

Doses are per infusion; /m^2^ and /kg denote cells per square metre of body surface area and per kilogram of body weight. Multivirus products targeted combinations of adenovirus, BKPyV, cytomegalovirus, Epstein–Barr virus, human herpesvirus 6 and JC polyomavirus; posoleucel targets six viruses. Abbreviations: BKPyV, BK polyomavirus; CD3+, cluster-of-differentiation-positive T cells; CMV, cytomegalovirus; HC, haemorrhagic cystitis; HCT, haematopoietic cell transplantation; HLA, human leukocyte antigen; IFN-γ, interferon gamma; IS, immunosuppression; JCPyV, JC polyomavirus; LT, large T antigen; mono, monospecific; NR, not reported; q14d/q28d/q4w, every 14 days/28 days/4 weeks; SOT, solid organ transplantation; VP1, viral capsid protein 1; VST, virus-specific T cell.

**Table 3:** BKPyV-specific efficacy-related outcomes by report.

Report	Response Definition (Time Point)	Responders, *n*/*N* (%)	Viral Load, Change	Clinical or Graft Outcome, *n*/*N* (%)	Follow-Up, mo
Papadopoulou 2014 [26]	CR or PR (NR)	5/6 (83.3); 4 CR, 1 PR	NR	Severe HC resolved 5/5 (100) responders	NR ^a^
Naik 2016 [27]	CR (NR)	1/2 (50.0)	NR; non-responder line lacked BK specificity	As Papadopoulou 2014 [26]	23 and 22
Pello 2017 [28]	HC resolution (day 20)	1/1 (100.0)	Urine 3.3 × 10^6^ → 1.4 × 10^3^ copies/mL (d 20) ^b^	HC resolved 1/1 (100)	2.5
Tzannou 2017 [29]	Clinical benefit (week 6)	16/16 (100.0)	Urine −85.5% (wk 6); −96% (wk 12), median	Haematuria resolved 13/14 (92.9); creatinine 1.70 → 1.25 mg/dL in 1/1 nephritis	3
Jahan 2020 [31]	Not defined (histology)	NA	Tissue SV40 30–50% → <1%	Graft loss 1/1 (100); fibrosis 80%	6
Rubinstein 2020 [33]	CR or PR (NR)	5/6 (83.3); per infusion 9/14 (64.3)	NR	Late response after reinfusion 3/4 (75.0)	NR
Nelson 2020 [34]	Response per infusion (4 weeks)	DNAemia 86%; HC 100%; both 87% (*N* NR)	NR	Reduction or clearance 3/3 (100) SOT	1 per infusion
Olson 2021 [35]	ORR, composite (days 14/28/45)	40/59 (67.8); 43/57 (75.4); 40/49 (81.6)	NR	Matched comparison favoured CTL recipients	12
Holland 2021 [36]	Not assessable	NA	Viraemia persisted after pre-HCT infusions	HC resolved 1/1 (100); death 1/1 (100), day +63	2
Kinoshita 2023 [37]	Cystitis resolution (NR)	1/1 (100.0)	Viruria persistent, down-trending	Cystitis resolved 1/1 (100)	5.8
Koldehoff 2023 [38]	Viral load decline (NR)	5/7 (71.4); immune response 6/7 (85.7)	NR	HC outcome incompletely reported	NR
Anand 2023 [39]	≥50% decline in BK load (week 12)	17/17 (100.0); *p* < 0.001	NR	Renal function not separable from IS reduction	8 (median)
Galletta 2023 [41]	Composite clinical response (NR)	27/48 (56.2)	NR	NR	NR
Pfeiffer 2023 [30]	PR, broad criteria (week 6)	27/27 (100.0)	NR	Haematuria/symptoms resolved 17/23 (73.9); nephritis recovery limited	1.5
Chandraker 2024 [42]	≥1-log and ≥50% plasma DNA decline (week 24)	≥1 log: 10/20 (50.0) q2w; 6/20 (30.0) q4w; 5/18 (27.8) placebo. ≥50%: 17/20 (85.0) q2w; 11/20 (55.0) q4w; 10/18 (55.6) placebo	NR	Graft endpoints exploratory; treatment-related graft loss 0	6
Khoury 2024 [40]	Virological response (NR)	45% (*N* NR)	NR	Renal recovery in BKPyVAN not demonstrated	NR
Neller 2024 [32]	Virological response (NR)	7/11 (63.6) evaluable; NA 5/16	NR	Clinical improvement 12/16 (75.0); end-organ response 7/10 (70.0)	NR
Ptak 2025 [43]	Transient clearance (12 months)	3/3 (100.0)	Mean 579 IU/mL (12 mo); 898 IU/mL (final)	Functional graft 2/3 (66.7); mean eGFR 37.3 → 33.5 mL/min/1.73 m^2^	12 (final 23.9)
Rubinstein 2025 [44]	CR or PR (4 weeks)	5/5 (100.0); 4 CR, 1 PR	NR	CR 4/5 (80.0) HC or DNAemia; PR 1/5 (20.0)	1
Olson 2026 [45]	CR or PR (day 45)	Kidney 5/6 (83.3); HCT 3/5 (60.0)	NR	Creatinine improvement 0/11 (0) consistent	12
Oberwegner 2026 [46]	Blood viral load reduction (day 60)	3/4 (75.0)	Log change −0.98, −0.73, −0.16, +0.46	HC cleared 1/1 (100); infection persisted 1/4 (25.0)	2
Schumacher 2026 [47]	Rapid BKPyV decline (NR)	1/1 (100.0)	CSF JCPyV cleared	Neurological symptoms resolved 1/1 (100); graft function reduced, stable	NR

^a^ The source classified seven BKPyV episodes, including one later post-infusion reactivation not counted here. ^b^ The figure legend of the source reported 1640 copies/mL. Follow-up is given in months, converted from the units used by the source; percentages are calculated only from stated numerators and denominators. Abbreviations: BK/BKPyV, BK polyomavirus; BKPyVAN, BKPyV-associated nephropathy; CR, complete response; CSF, cerebrospinal fluid; CTL, cytotoxic T lymphocyte; eGFR, estimated glomerular filtration rate; HC, haemorrhagic cystitis; HCT, haematopoietic cell transplantation; IS, immunosuppression; IU, international units; JCPyV, JC polyomavirus; NA, not applicable; NR, not reported; ORR, overall response rate; PR, partial response; q2w/q4w, every 2/4 weeks; SOT, solid organ transplantation; SV40, simian virus 40 immunostaining.

**Table 4:** Treatment-emergent safety observations by report.

Report	Denominator, *N* ^a^	Infusion Reaction or CRS, *n*/*N*	Acute GVHD, *n*/*N* (Grade)	Rejection, *n*/*N*	Other Events, *n*/*N*
Papadopoulou 2014 [26]	11 (whole cohort)	0/11 ^b^	1/11 (II, de novo)	NA	Bladder inflammation or pain 1/11
Naik 2016 [27]	2 (BK-treated)	0/2	0/2	NA	0/2
Pello 2017 [28]	1 (BK-treated)	0/1	0/1	NA	0/1
Tzannou 2017 [29]	38 (whole cohort)	0/38 (fever 1/38)	6/38 (5 I–II skin, 2 de novo; 1 III GI)	NA	NR
Jahan 2020 [31]	1 (BK-treated)	NR	NA	0/1	Graft nephrectomy 1/1
Rubinstein 2020 [33]	6 (BK-treated)	NR	NR	NA	NR
Nelson 2020 [34]	41 (whole cohort)	0/41	1/41 (stage 2 skin, recurrent)	NR	NR
Olson 2021 [35]	59 (BK-treated)	0/59	2/59 (1 II skin de novo; 1 III GI)	NA	Chronic GVHD 9/59 by 12 mo
Holland 2021 [36]	1 (BK-treated)	1/1 (severe, day 2 after 3rd infusion)	NR	NA	Death 1/1 day +63; SOS 1/1
Kinoshita 2023 [37]	1 (BK-treated)	0/1	0/1	NA	0/1
Koldehoff 2023 [38]	7 (BK-treated)	NR	NR	NA	Deaths 3/7 on longer follow-up
Anand 2023 [39]	17 (BK-treated)	0/17	0/17	1/17 (>1B TCMR)	MPGN 1/17, causality uncertain
Galletta 2023 [41]	77 donor-derived; 18 third-party (whole cohort)	NR	4/77 (5.2) vs. 2/18 (11.1), II–IV; *p* = 0.438	NA	NR
Pfeiffer 2023 [30]	58 (whole cohort)	0/58	13/58 (22.4) (mostly 1; distribution NR)	NA	NR
Chandraker 2024 [42]	61 (20 q2w; 22 q4w; 19 placebo)	0/61	0/61	3/42 posoleucel; 0/19 placebo	Any AE 50/61; treatment-related SAE 0/61; deaths 0/61
Khoury 2024 [40]	98 (whole cohort; 181 infusions)	0/98	NR	NR	NR
Neller 2024 [32]	78 (whole cohort)	0/78	0/78	0/78	NR
Ptak 2025 [43]	3 (BK-treated)	0/3	0/3	0/3	Grade 1 events 27; grade 2 events 7; SAE 0/3
Rubinstein 2025 [44]	5 (BK-treated)	0/5	0/5	NA	NR
Olson 2026 [45]	11 (6 kidney, 5 HCT)	0/11	0/5 (HCT)	0/6 (kidney)	NR
Oberwegner 2026 [46]	4 (BK-treated)	0/4	1/4 (II skin, de novo); 1/4 (GI, worsening)	NA	NR
Schumacher 2026 [47]	1 (BK-treated)	0/1	0/1	NR	IRIS 0/1

^a^ Denominator to which the safety statements refer: BK-treated, recipients treated for active BKPyV; whole cohort, all infused recipients, including those treated for other viral indications. ^b^ No infusion reaction or CRS; a local bladder reaction was reported. 0/*N* denotes an event explicitly reported as absent among *N* recipients; NA, not applicable to the transplant setting or not assessable; NR, not reported. Acute GVHD grades are given in parentheses. Abbreviations: AE, adverse event; BK, BK polyomavirus; CRS, cytokine release syndrome; GI, gastrointestinal; GVHD, graft-versus-host disease; HCT, haematopoietic cell transplantation; IRIS, immune reconstitution inflammatory syndrome; MPGN, membranoproliferative glomerulonephritis; q2w/q4w, every 2/4 weeks; SAE, serious adverse event; SOS, sinusoidal obstruction syndrome; TCMR, T-cell-mediated rejection.

**Table 5:** Virological response and organ-level outcome by BKPyV phenotype.

BKPyV Phenotype	Contributing Single-Phenotype Cohorts	Virological or Composite Response, *n*/*N* (%; 95% CI) ^a^	Organ-Level Outcome, *n*/*N* (%; 95% CI)	Mixed-Phenotype Reports Not Apportioned ^b^
DNAemia without organ disease	Chandraker 2024 [42] (posoleucel arms combined)	28/40 (70.0; 95% CI 55–82)	NA (no organ disease at entry)	Anand 2023 [39]; Rubinstein 2025 [44]; Ptak 2025 [43]; Nelson 2020 [34]
BKPyV-associated HC	Pello 2017 [28]; Olson 2021 [35]; Kinoshita 2023 [37]; Koldehoff 2023 [38]	47/68 (69.1; 95% CI 57–79)	Haematuria or cystitis resolved 19/25 (76.0; 95% CI 57–89) [28,30,37]	Papadopoulou 2014 [26]; Pfeiffer 2023 [30]; Galletta 2023 [41]; Oberwegner 2026 [46]
Biopsy-proven BKPyVAN	Olson 2026 [45]; Schumacher 2026 [47]	9/12 (75.0; 95% CI 47–91)	Consistent renal recovery 0/5 cohorts; graft lost 1/1 advanced case [31]	Pfeiffer 2023 [30] (2 nephritis cases); Khoury 2024 [40]; Neller 2024 [32]
Randomized comparator	Chandraker 2024 [42] placebo arm	10/18 (55.6; 95% CI 34–75)	NA	—

^a^ Each proportion uses the response definition of its source report without conversion: at least a 50% decline in plasma BKPyV DNA at week 24 for DNAemia; resolution of cystitis, or a composite of cystitis improvement and virological change, for HC; complete or partial response at day 45 for BKPyVAN. Intervals are Wilson intervals on the crude aggregate. ^b^ Cohorts enrolling more than one phenotype without a phenotype-specific numerator; counted in Figure 2 but not assignable to a phenotype. Abbreviations: BKPyV, BK polyomavirus; BKPyVAN, BKPyV-associated nephropathy; CI, confidence interval; HC, haemorrhagic cystitis.

**Table 6:** Proposed staged framework for considering BKPyV-reactive T-cell therapy within clinical trials.

Stage	Definition	Position of VST Therapy	Rationale
1. DNAemia, ROI not attempted	Plasma BKPyV DNA above threshold; no nephropathy on biopsy; IS not yet reduced	Not indicated; outside trial eligibility	ROI alone clears DNAemia in most recipients [6,8] and the trial placebo arm reached 10/18 (55.6%) at week 24 [42], so benefit cannot be shown
2. DNAemia persisting after ROI	Viral load not falling after staged ROI, or ROI limited by rejection risk; biopsy fibrosis absent or minimal	Preferred population for randomized trials	ROI has failed but injury is not yet fixed, so the virological endpoint stays measurable and meaningful; the phase I study enrolled this population [43]
3. BKPyVAN, moderate fibrosis	Established nephropathy; partially preserved function; incomplete interstitial fibrosis	Eligible with a graft-function endpoint, randomization stratified by fibrosis stage	Virological response remains achievable (9/12, 75.0%) but renal recovery was documented in no cohort [40,43,45]
4. BKPyVAN, advanced fibrosis	Advanced tubulointerstitial scarring; eGFR declining towards replacement therapy	Avoid outside mechanistic or palliative protocols	Antigen clearance does not reverse scarring: near-complete tissue clearance was followed by dialysis and nephrectomy with 80% fibrosis [31]
5. HC after HCT, refractory	Macroscopic haematuria with bladder pain despite hydration, transfusion and analgesia	Randomized trials only, with endpoints less prone to spontaneous resolution	Single-arm cohorts reported symptom resolution, but the phase III trial of time to haematuria resolution was stopped for futility (NCT04390113) [55]

Fibrosis stage refers to Banff interstitial fibrosis and tubular atrophy scoring [5]. This framework is a proposal for trial eligibility derived from the evidence synthesized in this review and is not a clinical treatment recommendation. Abbreviations: BKPyV, BK polyomavirus; BKPyVAN, BK polyomavirus-associated nephropathy; eGFR, estimated glomerular filtration rate; HC, haemorrhagic cystitis; HCT, haematopoietic cell transplantation; IS, immunosuppression; ROI, reduction in immunosuppression; VST, virus-specific T cell.

## Data Availability

Search strings, source yields, full-text exclusion reasons, report-linkage decisions, extracted results, appraisal judgments, and the cohort-level counts underlying Figure 2 and Figure 3 are reported in the manuscript, tables, figures, and appendices. No new data were created or analyzed in this study. Data sharing is not applicable to this article.

## Supplementary Materials

The following supporting information can be downloaded at: https://www.mdpi.com/article/10.3390/v18101166/s1. Supplementary Table S1: PRISMA-2020-Checklist.

## Appendix A. Reproducible Search Strategies

PubMed (180 records): ((“BK Virus”[Mesh] OR “BK polyomavirus”[tiab] OR “BK polyoma virus”[tiab] OR “polyomavirus BK”[tiab] OR “BK virus”[tiab] OR BKV[tiab] OR BKPyV[tiab] OR “human polyomavirus 1”[tiab]) AND (“Immunotherapy, Adoptive”[Mesh] OR “T-Lymphocytes, Cytotoxic”[Mesh] OR “adoptive immunotherapy”[tiab] OR “adoptive immunotherapies”[tiab] OR “adoptive cell therapy”[tiab] OR “adoptive T cell therapy”[tiab] OR “adoptive T-cell therapy”[tiab] OR “adoptive T-cell transfer”[tiab] OR “adoptive T cell transfer”[tiab] OR “adoptive transfer”[tiab] OR “virus-specific T cell”[tiab] OR “virus-specific T cells”[tiab] OR “virus-specific T-cell”[tiab] OR “virus-specific T-cells”[tiab] OR “virus specific T cell”[tiab] OR “virus specific T cells”[tiab] OR “BK-specific T cell”[tiab] OR “BK-specific T cells”[tiab] OR “BKV-specific T cell”[tiab] OR “BKV-specific T cells”[tiab] OR “BKPyV-specific T cell”[tiab] OR “BKPyV-specific T cells”[tiab] OR “cytotoxic T lymphocyte”[tiab] OR “cytotoxic T lymphocytes”[tiab] OR “cytotoxic T cell”[tiab] OR “cytotoxic T cells”[tiab] OR “multivirus-specific T cell”[tiab] OR “multivirus-specific T cells”[tiab] OR “multi-virus-specific T cell”[tiab] OR “multi-virus-specific T cells”[tiab] OR “third-party T cell”[tiab] OR “third-party T cells”[tiab] OR VST[tiab] OR VSTs[tiab] OR CTL[tiab] OR CTLs[tiab] OR posoleucel[tiab] OR ALVR105[tiab] OR “Viralym-M”[tiab]))

Europe PMC (73 records): (TITLE_ABS:“BK virus” OR TITLE_ABS:“BK polyomavirus” OR TITLE_ABS:BKPyV OR TITLE_ABS:BKV) AND (TITLE_ABS:“virus-specific T cell” OR TITLE_ABS:“virus specific T cell” OR TITLE_ABS:“cytotoxic T lymphocyte” OR TITLE_ABS:“adoptive T cell” OR TITLE_ABS:“adoptive immunotherapy” OR TITLE_ABS:posoleucel)

ClinicalTrials.gov (23 records): “BK virus” AND (“virus-specific T cells” OR posoleucel OR “cytotoxic T lymphocytes”)

Backward and forward citation checking of included reports and the most relevant reviews identified one additional eligible companion report (PMID 32697816). No automation tool excluded records.

## Appendix B. Full-Text Reports Excluded, with Reasons

Reports excluded at full text were: Wilhelm et al., an editorial without primary patient data [57]; Dadwal et al., scheduled post-HCT prophylaxis rather than treatment of active BKPyV [65]; Keller and Bollard, a review [68]; Dasari et al., no extractable outcome after therapeutic infusion [69]; Rubinstein et al., scheduled prophylactic VST administration [70]; Moss and Muller, a case report with a scoping review but no adoptive BKPyV-reactive T-cell infusion [71]; Al-Akioui Sanz et al., unselected familial CD45RA-negative memory T cells rather than a product selected for defined BKPyV specificity [72]; Gottlieb et al., prophylactic antigen-specific cells without therapeutic enrolment [73]; Lamarche et al., a manufacturing study without T-cell infusion [74]; and Green et al., a review of four studies all included here as primary reports [75].

## Appendix C. Study-Level Risk-of-Bias Judgments

Papadopoulou 2014 and Naik 2016 [26,27]: clear prospective patient-level reporting, but a small, uncontrolled and partly re-reported BK subgroup; high efficacy risk.

Pello 2017 [28]: intervention, chronology and outcome clear for one patient; very high selection, confounding and publication bias.

Tzannou 2017 and Pfeiffer 2023 [29,30]: prospective follow-up at prespecified times, but interim/expanded overlap, no comparator, repeat dosing, a broad composite response definition and missing evaluability; serious efficacy concerns.

Jahan 2020 [31]: detailed longitudinal biopsy and graft data, but a single case with multiple cointerventions and advanced baseline injury; very high efficacy risk.

Rubinstein 2020 and Nelson 2020 [33,34]: prospective infusion-level assessment with a defined 4-week response, but overlapping participants, repeat infusions, per-infusion denominators and no comparator; serious efficacy concerns.

Olson 2021 [35]: prospective ascertainment with a matched comparison, but nonrandom allocation, residual confounding, attrition across time points and a composite response; moderate-to-serious concerns.

Holland 2021 [36]: detailed safety chronology and competing causes; informative for signal detection but cannot estimate incidence or causality.

Kinoshita 2023 [37]: the eligible active-BK case was identifiable, but a single-case subgroup with coinfection and concurrent care; very high efficacy risk.

Koldehoff 2023 [38]: longitudinal immunomonitoring reported, but treatment selection, incomplete BK-subgroup outcomes and no comparator; serious concerns.

Anand 2023 [39]: defined kidney cohort with serial viral loads, but universal concomitant immunosuppression reduction, probable overlap with the expanded SOT cohort and no control group; serious confounding risk.

Galletta 2023 [41]: large retrospective comparison with a separable BK subgroup, but nonrandom product assignment, mixed viral indications, incomplete third-party safety follow-up and probable overlap; serious efficacy and safety concerns.

Chandraker 2024 [42]: randomized, blinded, placebo-controlled with objective viral load measurement, but later missing data and multiple exploratory endpoints; some concerns.

Khoury 2024 [40]: multicentre programme with broad applicability, but heterogeneous viruses and organs, overlap with earlier reports, immunosuppression changes and incomplete BK-specific detail; serious efficacy concerns.

Neller 2024 [32]: broad compassionate-access denominator with case-level outcomes, but retrospective selection, heterogeneous products and follow-up, missing fields and possible overlap; serious concerns.

Ptak 2025 [43]: protocolized phase I follow-up with explicit adverse-event reporting, but only 3 of 27 screened recipients treated and no comparator; high selection and efficacy risk, adequate for short-term safety.

Rubinstein 2025 [44]: prospective HLA-restriction methods and case-level viral loads, but a five-case subgroup with repeat dosing and no comparator; high efficacy risk.

Olson 2026 [45]: biopsy confirmation, transplant-stratified reporting and longitudinal follow-up, but small selected subgroups, repeat dosing, no comparator and advanced baseline damage; serious efficacy concerns.

Oberwegner 2026 [46]: patient-level dose, viral load, immune-expansion and GVHD data, but a four-case series with heterogeneous trajectories; very high efficacy risk.

Schumacher 2026 [47]: intervention and longitudinal virological and neurological course described, but a single coinfected case with repeat dosing and no comparator; very high efficacy risk.

## Appendix D. Cohorts and Definitions Used in the Quantitative Synthesis

Fifteen independent analytic cohorts contributed to Figure 2, with response definitions as given in Table 3: Papadopoulou 2014, 5 of 6 [26]; Pello 2017, 1 of 1 [28]; Olson 2021, 40 of 59 at day 14, the time point with the intact denominator [35]; Koldehoff 2023, 5 of 7 [38]; Kinoshita 2023, 1 of 1 [37]; Galletta 2023, 27 of 48 [41]; Pfeiffer 2023, 27 of 27 [30]; Rubinstein 2025, 5 of 5 [44]; Oberwegner 2026, 3 of 4 [46]; Anand 2023, 17 of 17 [39]; Chandraker 2024, 28 of 40 with the posoleucel arms combined and the placebo arm (10 of 18) displayed separately [42]; Ptak 2025, 3 of 3 [43]; Schumacher 2026, 1 of 1 [47]; Olson 2026, 8 of 11 (5 of 6 kidney, 3 of 5 HCT), split by transplant type in Figure 2 and counted once [45]; and Neller 2024, 7 of 11 [32]. Weights ranged from 2.3% for each single-patient report to 10.9% for the largest cohort. The eight cohorts reporting only a combined numerator across phenotypes, and therefore not apportioned in Table 5, were 5 of 6 [26], 27 of 27 [30], 17 of 17 [39], 27 of 48 [41], 7 of 11 [32], 5 of 5 [44], 3 of 4 [46] and 3 of 3 [43].

Seven included reports were excluded from Figure 2 to preserve independence or because no usable denominator was available: Naik 2016 [27], whose two BK cases were already reported by Papadopoulou; Tzannou 2017 [29], nested in Pfeiffer; Rubinstein 2020 [33], overlapping Nelson; Nelson 2020 [34], which reported response per infusion, the Cincinnati HCT experience being represented by Galletta; Holland 2021 [36], a single safety case from the Nelson protocol; Jahan 2020 [31], a probable contributor to Neller; and Khoury 2024 [40], whose BK-specific denominator was not reported and whose solid organ experience is represented by Anand. This rule is stricter than the report-linkage count in Section 3.1.

Figure 3 used the same independence rules applied to allogeneic HCT reports with a stated acute GVHD numerator and denominator (Table 4). Nelson 2020 [34] was represented by Galletta 2023 [41] because of probable overlap, and Tzannou 2017 [29] by Pfeiffer 2023 [30]. The Galletta donor-derived and third-party groups were displayed separately as reported and were not summed.
