# Supplementary material for: Efficacy and Safety of Adoptive BK Polyomavirus-Reactive T-Cell Therapy for Active Infection or Disease in Transplant Recipients: A Systematic Review
Source: Viruses. 2026 Oct 20;18(10):1166. doi: 10.3390/v18101166 (PMC13709701; doi:10.3390/v18101166)
Supplement: Supplementary file 1 [file viruses-18-01166-s001.zip › viruses-4576596-supplementary.pdf]

PRISMA 2020 Checklist

Completed for viruses-4576596 | 6 September 2026
Efficacy and Safety of Adoptive BK Polyomavirus–Reactive T-Cell Therapy for Active Infection or Disease in Transplant Recipients: A Systematic Review
Locations refer to the supplied manuscript. Section references are definitive; page numbers follow its rendered 27-page layout. Partial or unreported elements are identified explicitly; completing this checklist does not certify full compliance.

Section and Topic	Item #	Checklist item	Location where item is reported
**TITLE**			
Title	1	Identify the report as a systematic review.	Title, p. 1: explicitly identifies a systematic review.
**ABSTRACT**			
Abstract	2	See the PRISMA 2020 for Abstracts checklist.	Abstract, p. 1. Partial: objectives, eligibility, sources/date, synthesis, findings, limitations and interpretation are reported. Risk-of-bias methods, review funding and registration are not included in the abstract.
**INTRODUCTION**			
Rationale	3	Describe the rationale for the review in the context of existing knowledge.	Section 1, Introduction, pp. 2–3.
Objectives	4	Provide an explicit statement of the objective(s) or question(s) the review addresses.	Section 1, final paragraph, p. 3; Section 2.1 (PICO), pp. 3–4.
**METHODS**			
Eligibility criteria	5	Specify the inclusion and exclusion criteria for the review and how studies were grouped for the syntheses.	Sections 2.1–2.2, pp. 3–4; Section 2.5, pp. 5–6 (grouping by transplant setting and phenotype).
Information sources	6	Specify all databases, registers, websites, organisations, reference lists and other sources searched or consulted to identify studies. Specify the date when each source was last searched or consulted.	Section 2.3, pp. 4–5; Appendix A, p. 20. Database/register cutoff: 1 September 2026. Separate dates for citation checking and publisher-page consultation are not specified.
Search strategy	7	Present the full search strategies for all databases, registers and websites, including any filters and limits used.	Section 2.3, pp. 4–5; Appendix A, p. 20 (PubMed, Europe PMC, ClinicalTrials.gov queries; no filters).
Selection process	8	Specify the methods used to decide whether a study met the inclusion criteria of the review, including how many reviewers screened each record and each report retrieved, whether they worked independently, and if applicable, details of automation tools used in the process.	Section 2.4, p. 5: single-reviewer screening with a second source-level audit, not dual-independent screening. Appendix A, p. 20: no automated exclusions.
Data collection process	9	Specify the methods used to collect data from reports, including how many reviewers collected data from each report, whether they worked independently, any processes for obtaining or confirming data from study investigators, and if applicable, details of automation tools used in the process.	Sections 2.1 and 2.4, pp. 3–5; Section 5, p. 19. Single-reviewer extraction, second source-level audit; no author contact or patient-level data requests.
Data items	10a	List and define all outcomes for which data were sought. Specify whether all results that were compatible with each outcome domain in each study were sought (e.g. for all measures, time points, analyses), and if not, the methods used to decide which results to collect.	Sections 2.1, 2.4–2.5, pp. 3–6; Table 3, pp. 11–12; Appendix D, pp. 22–23. Outcome domains and time-point handling are described; comprehensive within-study result-selection rules are not fully specified.
	10b	List and define all other variables for which data were sought (e.g. participant and intervention characteristics, funding sources). Describe any assumptions made about any missing or unclear information.	Sections 2.1 and 2.4–2.5, pp. 3–6; Tables 1–2, pp. 7–10. Missing values recorded as NR; no imputation or reconstruction of unreported denominators.
Study risk of bias assessment	11	Specify the methods used to assess risk of bias in the included studies, including details of the tool(s) used, how many reviewers assessed each study and whether they worked independently, and if applicable, details of automation tools used in the process.	Section 2.5, pp. 5–6; Appendix C, pp. 21–22. Partial: RoB 2 and JBI domains described; number and independence of risk-of-bias assessors are not specified.
Effect measures	12	Specify for each outcome the effect measure(s) (e.g. risk ratio, mean difference) used in the synthesis or presentation of results.	Section 2.5, pp. 5–6; Tables 3–4, pp. 11–14; Figures 2–3, pp. 15–16. Response and GVHD proportions with Wilson 95% CIs; no pooled comparative treatment effect.

PRISMA 2020 Checklist

Section and Topic	Item #	Checklist item	Location where item is reported
Synthesis methods	13a	Describe the processes used to decide which studies were eligible for each synthesis (e.g. tabulating the study intervention characteristics and comparing against the planned groups for each synthesis (item #5)).	Sections 2.4–2.5, pp. 5–6; Appendix D, pp. 22–23: cohort linkage, overlap exclusions and denominator requirements.
	13b	Describe any methods required to prepare the data for presentation or synthesis, such as handling of missing summary statistics, or data conversions.	Sections 2.1, 2.4–2.5, pp. 3–6; Appendix D, pp. 22–23. Missing data, transformations, combined trial arms and response definitions described.
	13c	Describe any methods used to tabulate or visually display results of individual studies and syntheses.	Section 2.5, pp. 5–6; Tables 1–4, pp. 7–14; Figures 2–3, pp. 15–16; Appendix D, pp. 22–23.
	13d	Describe any methods used to synthesize results and provide a rationale for the choice(s). If meta-analysis was performed, describe the model(s), method(s) to identify the presence and extent of statistical heterogeneity, and software package(s) used.	Sections 2.1 and 2.5, pp. 3–6. Narrative synthesis and exploratory random-effects proportions; Freeman–Tukey transformation, DerSimonian–Laird variance, Q and I^2 specified. Analysis software/package is not reported.
	13e	Describe any methods used to explore possible causes of heterogeneity among study results (e.g. subgroup analysis, meta-regression).	Section 2.5, pp. 5–6: no subgroup meta-analysis. Section 4, pp. 17–18: qualitative discussion of heterogeneity; no meta-regression reported.
	13f	Describe any sensitivity analyses conducted to assess robustness of the synthesized results.	Section 2.5, p. 6: sensitivity analysis restricted to cohorts with at least five evaluable recipients.
Reporting bias assessment	14	Describe any methods used to assess risk of bias due to missing results in a synthesis (arising from reporting biases).	Section 2.5, p. 6: small-study-effect tests not applied. Sections 3.7 and 5 discuss publication/reporting concerns; no structured synthesis-level missing-results assessment method is reported.
Certainty assessment	15	Describe any methods used to assess certainty (or confidence) in the body of evidence for an outcome.	Section 2.5, p. 6: qualitative assessment considers risk of bias, inconsistency, indirectness, imprecision and comparative evidence.
**RESULTS**			
Study selection	16a	Describe the results of the search and selection process, from the number of records identified in the search to the number of studies included in the review, ideally using a flow diagram.	Section 3.1, p. 6; Figure 1, p. 7. Flow: 276 database/register records; 73 duplicates; 203 screened; 30 full texts; 9 excluded; 1 citation-search addition; 22 included reports.
	16b	Cite studies that might appear to meet the inclusion criteria, but which were excluded, and explain why they were excluded.	Section 3.1, p. 6; Appendix B, pp. 20–21 (nine cited exclusions with reasons).
Study characteristics	17	Cite each included study and present its characteristics.	Sections 3.2–3.3, pp. 7–11; Tables 1–2; references [33–54].
Risk of bias in studies	18	Present assessments of risk of bias for each included study.	Section 3.7, pp. 16–17; Appendix C, pp. 21–22. Narrative study-level judgments provided; complete tool-domain matrices are not presented.
Results of individual studies	19	For all outcomes, present, for each study: (a) summary statistics for each group (where appropriate) and (b) an effect estimate and its precision (e.g. confidence/credible interval), ideally using structured tables or plots.	Sections 3.4–3.6, pp. 11–16; Tables 3–4; Figures 2–3; Appendix D. Study results and available denominators; CIs for plotted proportions; unavailable results marked NR.
Results of syntheses	20a	For each synthesis, briefly summarise the characteristics and risk of bias among contributing studies.	Sections 3.2–3.7, pp. 7–17; Tables 1–4; Appendix C and Appendix D. Characteristics and bias are summarized across the contributing evidence.
	20b	Present results of all statistical syntheses conducted. If meta-analysis was done, present for each the summary estimate and its precision (e.g. confidence/credible interval) and measures of statistical heterogeneity. If comparing groups, describe the direction of the effect.	Section 3.6, pp. 14–16; Figure 2: exploratory pooled response 87% (95% CI 73–97), I^2 = 70%; study-level GVHD in Figure 3 without pooling.

PRISMA

PRISMA 2020 Checklist

Section and Topic	Item #	Checklist item	Location where item is reported
	20c	Present results of all investigations of possible causes of heterogeneity among study results.	Section 4, pp. 17–18: qualitative explanations of heterogeneity. No formal subgroup or meta-regression results, as those analyses were not performed.
	20d	Present results of all sensitivity analyses conducted to assess the robustness of the synthesized results.	Section 3.6, pp. 14–15; Section 4, pp. 17–18: restricted analysis 82% (95% CI 67–93) in ten cohorts with at least five evaluable recipients.
Reporting biases	21	Present assessments of risk of bias due to missing results (arising from reporting biases) for each synthesis assessed.	Sections 3.7, 4 and 5, pp. 16–19: qualitative reporting/publication-bias concerns. No formal assessment for each synthesis is reported.
Certainty of evidence	22	Present assessments of certainty (or confidence) in the body of evidence for each outcome assessed.	Section 3.7, pp. 16–17: low certainty for randomized virological comparison; very low for symptom, graft, survival and rare safety outcomes. Qualitative judgments, not a formal certainty table.
**DISCUSSION**			
Discussion	23a	Provide a general interpretation of the results in the context of other evidence.	Section 4, Discussion, pp. 17–19.
	23b	Discuss any limitations of the evidence included in the review.	Section 5, Limitations, p. 19; Section 4, pp. 17–19.
	23c	Discuss any limitations of the review processes used.	Section 5, p. 19: database coverage, full-text availability, single-reviewer process, unresolved overlap and absence of author contact.
	23d	Discuss implications of the results for practice, policy, and future research.	Section 4, final paragraph, pp. 18–19; Section 6, p. 19.
**OTHER INFORMATION**			
Registration and protocol	24a	Provide registration information for the review, including register name and registration number, or state that the review was not registered.	Section 2.1, p. 3: Open Science Framework, osf.io/zcwx6 (as stated in the manuscript).
	24b	Indicate where the review protocol can be accessed, or state that a protocol was not prepared.	Section 2.1, pp. 3–4: OSF identifier and protocol content described. Public accessibility and registered-protocol status were not independently verified for this checklist.
	24c	Describe and explain any amendments to information provided at registration or in the protocol.	Section 2.1, pp. 3–4: two protocol departures described (prophylaxis-only exclusion; additional therapeutic cohorts/companion reports).
Support	25	Describe sources of financial or non-financial support for the review, and the role of the funders or sponsors in the review.	Funding statement, p. 20: no external research funding; APC paid by UMFVBT. The APC funder's role and non-financial support are not explicitly described.
Competing interests	26	Declare any competing interests of review authors.	Conflicts of Interest statement, p. 20: no conflicts declared.
Availability of data, code and other materials	27	Report which of the following are publicly available and where they can be found: template data collection forms; data extracted from included studies; data used for all analyses; analytic code; any other materials used in the review.	Data Availability Statement, p. 20; Tables 1–4; Appendices A–D. Extracted results and analysis counts are provided within the manuscript. Analytic code and blank extraction-form availability are not specified.

From: Page MJ, McKenzie JE, Bossuyt PM, Boutron I, Hoffmann TC, Mulrow CD, et al. The PRISMA 2020 statement: an updated guideline for reporting systematic reviews. BMJ 2021;372:n71. doi: 10.1136/bmj.n71. This work is licensed under CC BY 4.0. To view a copy of this license, visit https://creativecommons.org/licenses/by/4.0/
